# Nitrated monoaromatic hydrocarbons (nitrophenols, nitrocatechols, nitrosalicylic acids) in ambient air: levels, mass size distributions and inhalation bioaccessibility

**DOI:** 10.1007/s11356-020-09540-3

**Published:** 2020-06-11

**Authors:** Zoran Kitanovski, Jan Hovorka, Jan Kuta, Cecilia Leoni, Roman Prokeš, Ondřej Sáňka, Pourya Shahpoury, Gerhard Lammel

**Affiliations:** 1 Multiphase Chemistry Department, Max Planck Institute for Chemistry, Mainz, Germany; 2 Institute for Environmental Studies, Faculty of Science, Charles University, Prague, Czech Republic; 3 Research Centre for Toxic Compounds in the Environment, Masaryk University, Brno, Czech Republic; 4 Air Quality Processes Research Section, Environment and Climate Change Canada, Toronto, Canada

**Keywords:** Air pollution, Nitroaromatic compounds, Bioaccessibility, Aerosol

## Abstract

Nitrated monoaromatic hydrocarbons (NMAHs) are ubiquitous in the environment and an important part of atmospheric humic-like substances (HULIS) and brown carbon. They are ecotoxic and with underresearched toxic potential for humans. NMAHs were determined in size-segregated ambient particulate matter collected at two urban sites in central Europe, Ostrava and Kladno, Czech Republic. The average sums of 12 NMAHs (Σ_12_NMAH) measured in winter PM_10_ samples from Ostrava and Kladno were 102 and 93 ng m^−3^, respectively, and 8.8 ng m^−3^ in summer PM_10_ samples from Ostrava. The concentrations in winter corresponded to 6.3–7.3% and 2.6–3.1% of HULIS-C and water-soluble organic carbon (WSOC), respectively. Nitrocatechols represented 67–93%, 61–73% and 28–96% of NMAHs in PM_10_ samples collected in winter and summer at Ostrava and in winter at Kladno, respectively. The mass size distribution of the targeted substance classes peaked in the submicrometre size fractions (PM_1_), often in the PM_0.5_ size fraction especially in summer. The bioaccessible fraction of NMAHs was determined by leaching PM_3_ samples in two simulated lung fluids, Gamble’s solution and artificial lysosomal fluid (ALF). More than half of NMAH mass is found bioaccessible, almost complete for nitrosalicylic acids. The bioaccessible fraction was generally higher when using ALF (mimics the chemical environment created by macrophage activity, pH 4.5) than Gamble’s solution (pH 7.4). Bioaccessibility may be negligible for lipophilic substances (i.e. log *K*_OW_ > 4.5).

**Electronic supplementary material:**

The online version of this article (10.1007/s11356-020-09540-3) contains supplementary material, which is available to authorized users.

## Introduction

Nitrated monoaromatic hydrocarbons (NMAHs) are an important part of humic-like substances (HULIS), which in turn constitute a large mass fraction of particulate matter (PM) water-soluble organic carbon (WSOC; Graber and Rudich 2006) and brown carbon (Laskin et al. 2015). NMAHs are primarily emitted into the atmosphere or formed by secondary processes. Gas- and aqueous-phase oxidation and nitration of lignin thermal decomposition products (m-cresol, phenols, methoxyphenols, catechols, salicylic acid, etc.) are major formation pathways for 4-nitrocatechol (4-NC), methylnitrocatechols (MNCs), nitroguaiacols (NGs) and nitrosalicylic acids (NSAs; Iinuma et al. 2010; Kelly et al. 2010; Kroflič et al. 2015; Frka et al. 2016; Teich et al. 2017; Xie et al. 2017; Finewax et al. 2018; Wang et al. 2019). Traffic and coal and wood combustion, as well as industry and agricultural use of pesticides, are considered as main primary emission sources of nitrophenols (NPs), which can also be secondarily formed in the atmosphere (Harrison et al. 2005; Iinuma et al. 2007; Kitanovski et al. 2012; Inomata et al. 2015; Wang et al. 2018). 4-NC and MNCs are well-established tracers for biomass burning secondary organic aerosols (Iinuma et al. 2010; Kitanovski et al. 2012; Kahnt et al. 2013; Caumo et al. 2016; Chow et al. 2016). NSAs may also be formed in secondary organic aerosols exposed to NO_3_ radicals (Ramaswamy et al. 2019). NMAHs may represent up to 1% and 2% of PM_10_ mass and HULIS, respectively (Claeys et al. 2012; Kitanovski et al. 2012, 2020; Kahnt et al. 2013; Caumo et al. 2016). NPs and NSAs are proven to have adverse effects on human health (estrogenic activity, carcinogenicity, cataract; Karim and Gupta 2001; Brüning et al. 2002; Harrison et al. 2005; Grundlingh et al. 2011; Kovacic and Somanathan 2014), while little is known about the toxicology of NCs. NMAHs may redox cycle in epithelial lung fluid and be a source of reactive oxygen species (ROS) in the lungs.

Organic chemicals in ambient PM contribute significantly to air pollution and its adverse health effects (Lewtas 1993; Jones 1999; Shiraiwa et al. 2017). Extracts of ambient wood burning aerosol induce mutagenicity and intracellular production of ROS more than road traffic aerosol (Velali et al. 2019). Polar fractions of organic PM extracts show higher toxicities than apolar ones (Nováková et al. 2020). The complete pollutant mass in the air may not be bioaccessible upon inhalation as the dissolution of the substance in the epithelial lung lining fluid (LLF) is a prerequisite for biological activity. However, this prerequisite is not needed, when the substances are carried by ultrafine particles which may penetrate membranes completely (Oberdörster et al. 2004; Li et al. 2017). Unlike heavy metals in PM (Wiseman and Zereini 2014; Wiseman 2015; Kastury et al. 2017; Polezer et al. 2019), the organic matter (OM) fraction of PM that is potentially soluble in LLF has hardly been studied. The most common approach for in vitro assessment of the bioaccessibility of PM chemicals in LLF is by determining the fraction of the total concentration of a chemical leached from PM deposited filters immersed in simulated LLFs, under controlled conditions (Wiseman 2015). The two most commonly used simulated LLFs are artificial lysosomal fluid (ALF; Colombo et al. 2008; Wiseman 2015) and Gamble’s solution (Marques et al. 2011; Wiseman 2015). ALF mimics the chemical environment around inhaled particles after being phagocytized by lung alveolar and interstitial macrophages. It is an acidic aqueous electrolyte without lipids, pH 4.5 (Table S1). Gamble’s solution is the most common simulated LLF and represents the interstitial fluid in the lung. It is a neutral aqueous electrolyte without lipids, proteins and antioxidants, pH 7.4 (Table S1). The bioaccessible fraction of a chemical in PM is calculated as *f*_bio_p_ = *c*_p LLF_/*c*_p MeOH_ × 100 (%), where *c*_p LLF_ is the leached concentration in LLF and *c*_p MeOH_ is the total concentration (from extraction in methanol) of the substance in PM samples used for leaching.

The aim of this present study was to determine levels and mass size distributions of NMAHs in the atmospheric PM collected at two urban locations in the Czech Republic. Inhalation bioaccessibility of semivolatile organic compounds so far has been mostly focusing on PAHs (Wei et al. 2018). For the first time, we quantify the inhalation bioaccessibility of NMAHs in PM. PAHs’ and nitro- and oxy-PAHs’ abundances and bioaccessibility in the same PM samples (Lammel et al. 2020a, b), as well as simultaneously in the gas phase (Lammel et al. 2020a), are presented in companion papers. Toxicities of these PM samples, as well as the mixture toxicity of the substance classes addressed (reconstituted mixtures), are published elsewhere (Nováková et al. 2020).

## Materials and methods

### Sampling sites

Air samples were collected at two urban and one rural site in the Czech Republic, Kladno-Švermov (50° 10′ 01″ N/14° 06′ 15″E) during 10–14 February 2016 and Ostrava-Přivoz (49°51′23″N/18°16′11″E) during 15–27 February and 5–17 September 2016, respectively (Fig. S1). In Kladno, an industrial town (≈ 70,000 inhabitants), a heat plant but no major industries were working during the campaign. The Ostrava site is located quite central in the industrial area (≈ 500,000 inhabitants). It is a station of the Czech Hydrometeorological Institute (CHMI). A major cokery with 200 furnaces, a major metallurgical plant, a waste burner and other industries are within 3 km from the site. Ostrava is a hot spot of air pollution in Europe (Pokorná et al. 2015, 2016; Kozáková et al. 2019). For example, abundance of polycyclic aromatic hydrocarbons (PAHs) is high in Ostrava and the biological effects of PM are evident, in particular during winter time (Líbalová et al. 2012; Šram et al. 2013; Topinka et al. 2015; Pokorná et al. 2015; Leoni et al. 2016).

### Sampling

Particulate and gas phase samples were collected side by side by a high-volume air sampler Digitel DH77 (Digitel, Hegnau, Switzerland) and a high-volume 6-stage slot impactor Baghirra HV-100P (Baghirra, Prague). All samplers had PM_10_ inlets. Only the particulate phase samples were used for this study, i.e. 6 and 12 Digitel samples collected at Kladno and Ostrava winter campaigns, respectively, and 1, 3 and 3 impactor samples collected at Kladno and Ostrava winter and summer campaigns, respectively. The Digitel sampler was equipped with a quartz fibre filter (QFF, Whatman, Little Chalfont, UK), and the Baghirra sampler equipped with a multi-stage cascade impactor (Tisch Environmental Inc., Cleves, USA, series 230, model 235) with five impactor stages, corresponding to 10–7.2, 7.2–3, 3–1.5, 1.5–0.95 and 0.95–0.49 μm of aerodynamic particle size, *D*, (spaced roughly equal Δlog*D*) and a backup filter collecting particles < 0.49 μm. In the impactor, PM was collected on a slotted quartz fibre filter (QFFs, TE-230-QZ, Tisch Environmental Inc., Cleves, USA, 14.3 × 13.7 cm) and the backup filter was a QFF (Whatman). The samplers were operated at constant flow rates of ≈ 29 (Digitel, 24 h sampling) and ≈ 68 m^3^ h^−1^ (Baghirra, 96-h sampling).

Filter samples were kept on-site and during transport cool (≈ 0 °C), then stored at temperatures below − 18 °C.

### Leaching of NMAHs in simulated lung fluids and chemical analysis

Two LLFs were used, i.e. artificial lysosomal fluid (ALF; Colombo et al. 2008) and Gamble’s solution (Marques et al. 2011). Their compositions are given in the supplementary material (SM) (Table S1). The bioaccessible fractions of NMAHs in PM_3_ (*f*_bio_) were obtained by leaching the slotted and backup PM deposited QFFs with particles < 3 μm in 20 mL of simulated LLF by shaking (60 revolutions min^−1^) in a 100-mL flask during 24 h in an incubator at 37 °C, in the dark. Dependent on NMAH load, 1.5-cm^2^ cuts up to one strip (out of 10 strips of length 12 cm) of each slotted QFF were leached, while 1.5–20-cm^2^ cuts were leached from backup QFFs. The leachates were filtered through 0.45-μm cellulose acetate membrane, acidified with formic acid (1.0 mL 98–100% formic acid per 20 mL leachate), spiked with 4-nitrophenol-d_4_ (internal standard (IS); spiked mass 100 ng) and loaded on solid-phase extraction disks (SPE disks; BakerBond SPEEDISK DVB H_2_Ophilic, J.T. Baker). Targeted compounds were eluted from SPE disks sequentially with methanolic solution of EDTA (3.4 nmol mL^−1^) and a mixture of methanolic solution of EDTA (3.4 nmol mL^−1^) and acetonitrile (1:1). The obtained extract was concentrated to 0.5 mL using a TurboVap II (bath temperature, 40 °C; nitrogen gas pressure, 15 psi; Biotage, Uppsala, Sweden). The concentrated extract was filtered through a 0.2-μm PTFE syringe filter (4 mm, Whatman; GE Healthcare, Little Chalfont, UK) into a 2-mL vial and was evaporated to near dryness under the gentle stream of nitrogen (99.999%; Westfalen AG, Münster, Germany). Finally, the extract was dissolved in methanol/water mixture (3/7, v/v) containing 5 mM ammonium formate buffer pH 3 and 400 μM EDTA for LC/MS analysis.

The SPE recoveries of NMAHs were 94–101% in methanol extracts, 95–105% and 95–104% from ALF and Gamble’s solution leachates, respectively (Table S3). The results were not recovery corrected.

The determination of NMAHs in the PM filter samples was done using a validated analytical procedure (Kitanovski et al. 2012, 2020) with small modifications. In short, a 1.5-cm^2^ section of the filter was spiked with 4-nitrophenol-d_4_ (IS; spiked mass, 100 ng) and extracted three times (5 min each) with 10 mL methanolic solution of EDTA (3.4 nmol mL^−1^) in an ultrasonic bath. The combined extracts were concentrated, filtered, dried and re-dissolved for LC/MS analysis as described above for SPE extracts.

The targeted NMAHs, i.e. 2 NSAs, 4 NCs and 6 NPs (listed in Table S2 together with main physicochemical properties), were determined using an Agilent 1200 Series HPLC system (Agilent Technologies, Waldbronn, Germany) coupled to an Agilent 6130B single quadrupole mass spectrometer equipped with an electrospray ionisation (ESI) source (Kitanovski et al. 2020). Atlantis T3 column (150 mm × 2.1 mm i.d., 3-μm particles size; Waters, Milford, USA), connected to an Atlantis T3 VanGuard pre-column (5 mm × 2.1 mm i.d., 3-μm particles size; Waters), was used for the separation of the targeted analytes. NMAHs were eluted isocratically using a mobile phase consisted of methanol/tetrahydrofuran/water (30/15/55, v/v/v) mixture containing 5 mM ammonium formate buffer pH 3 at a flow rate of 0.2 mL min^−1^. The column temperature and injection volume were 30 °C and 10 μL, respectively (Kitanovski et al. 2012). For the detection and quantification of NMAHs, the mass spectrometer was operated in single ion monitoring (SIM) and negative ESI mode. The optimised ESI-MS parameters were as follows: 1000 V for the ESI capillary voltage, 30 psig for the nebulizer pressure and 12 L min^−1^ and 340 °C for the drying gas flow and temperature, respectively. High-purity nitrogen was used as a nebulizer and drying gas. 3-Methyl-4-nitrocatechol (3-M-4-NC) concentrations were calculated based on the calibration curve of 4-methyl-5-nitrocatechol (4-M-5-NC) due to the lack of a reference standard for 3-M-4-NC and its structural similarity to 4-M-5-NC. LC/MSD ChemStation (Agilent Technologies) was used for data acquisition and analysis.

Field blanks (*n* = 3) were prepared during sample collection by mounting the pre-baked filters on the sampler without switching it on. These filters were subsequently retrieved and processed along with the rest of the samples. The mean of two or three field blank values was subtracted from the sample values (in both methanol extracts and leachates). Values below the mean + 3 standard deviations of the field blank values were considered to be below the limit of quantification (<LOQ). LOQs for the various campaigns are listed in Table S4. Heavy metal content, aerosol number and mass size distributions (MSDs), meteorological and trace gases were also covered by respective methods, described in the supplementary material (SM) (S1.4).

## Results and discussion

### Concentration levels and mass size distributions

The levels of the targeted substance classes in PM_10_ are listed in Table 1, and the time series are shown in Fig. S2. With PM_2.5_ ranging 15–34 μg m^−3^ (Table 1), the sites were considerably polluted. The pollution by heavy metals in Ostrava air was found very high, independently of season (Table 1; Fig. S3) and must be seen in the context of the local metallurgical industries and coal production and burning (Pokorná et al. 2015; Vossler et al. 2015). The pollution at the urban sites was less reflected by the levels of the secondary inorganic aerosol (SO_4_^2−^, NO_3_^−^, NH_4_^+^), because these are regionally distributed pollutants, exhibiting a low urban-to-rural gradient (Lammel et al. 2003).

**Table 1:** NMAH concentration and (a) mass mixing ratio (in PM_10_ (ppmm)), ambient PM mass concentrations (PM_10_, PM_2.5_) and chemical composition, inorganic gaseous pollutants and meteorological data, and (b) individual NMAHs and substance classes in PM_10_/PM_3_

	Kladno winter	Ostrava winter	Ostrava summer
(a)			
Σ_12_NMAH (ng m^−3^) (Σ_12_NMAH (ppmm))	93 (4940)	102 (2519)	8.8 (205)
PM_10_/PM_2.5_ (μg m^−3^)	16.9/15.4	39.9/34.2	40.2/30.1
Σ_10_OPAH (ng m^−3^) (Σ_10_OPAH (ppmm))^a^	5.6 (380)	10.0 (250)	4.0 (99)
HULIS-C (μg m^−3^)^b^	1.47	1.39	n.d.
WSOC (μg m^−3^)^b^	3.62	3.30	n.d.
EC/OC (μg m^−3^)	0.9/6.9	1.4/7.1	1.4/6.0
NO_x_/CO (ppbv)	15.4/320	45.1/496	41.3/375
SO_4_^2−^/NO_3_^−^/NH_4_^+^ (μg m^−3^)^b^	1.1/3.9/1.8	2.2/4.2/3.2	n.d.
Fe/Pb (ng m^−3^)	186/6.8	977/21	1285/20
Temperature (°C)	0.9 (− 6–10)	4.1 (− 4–15)	20 (11–29)
Rel. humidity (%)	79 (47–95)	80 (41–97)	70 (35–95)
(b) Analyte			
3-NSA	0.30/0.28	0.37/0.33	0.32/0.31
5-NSA	0.50/0.44	0.73/0.62	0.79/0.73
Σ_2_NSA	0.80/0.72	1.10/0.95	1.11/1.04
4-M-5-NC	20.5/19.9	21.9/21.2	1.44/1.44
3-M-5-NC	14.6/14.2	14.49/13.98	0.70/0.70
3-M-4-NC	3.77/3.67	3.79/3.68	0.19/0.23
4-NC	42.8/41.5	38.3/37.2	3.89/3.88
Σ_4_NC	81.8/79.3	78.4/76.1	6.27/6.24
4-NP	5.54/3.01	9.06/5.35	1.12/0.85
4-NG	< 0.061/< 0.061	1.23/0.38	< 0.026/< 0.026
DNOC	< 0.006/< 0.006	0.034/< 0.006	< 0.0065/< 0.0065
2,4-DNP	< 0.006/< 0.006	0.14/0.077	0.0088/< 0.0065
3-M-4-NP	2.40/1.43	5.45/3.58	0.15/0.15
2-M-4-NP	2.91/1.81	7.24/4.97	0.26/0.26
Σ_6_NP	10.9/6.24	21.9/14.0	1.64/1.26
Σ_12_NMAH	93.4/86.2	101.8/91.1	8.84/8.37

*OPAH*, oxygenated polycyclic aromatic hydrocarbons; *HULIS-C*, humic-like substances; *WSOC*, water-soluble organic carbon; *n.d.*, no data

^a^Lammel et al. (2020b)

^b^Voliotis et al. (2017)

The NMAH levels at the Kladno and Ostrava (winter) sites corresponded to 2.6 and 3.1% of the WSOC, respectively, and 6.3 and 7.3% of the HULIS-C, respectively (Voliotis et al. 2017). NMAHs were dominated by 4-NC and MNCs (Fig. S4a). The patterns in PM_1_ and PM_10_ are rather similar unlike typical for many other aerosol constituents (Putaud et al. 2010).

**Fig. 1:**
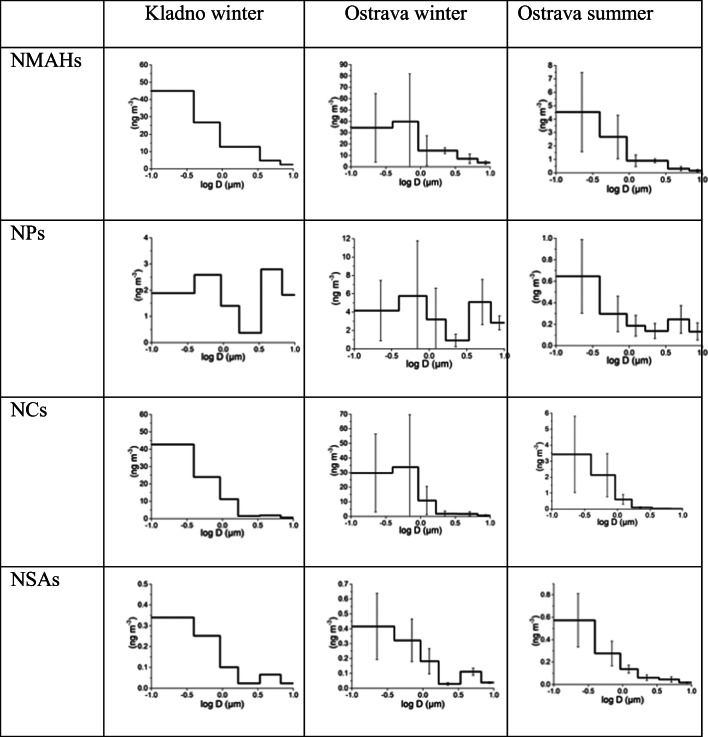
Time-weighted mean Σ_12_NMAHs and sub-classes’ mass size distributions. The error bars show the standard deviation from the campaign mean (*n* = 3 for Ostrava, *n* = 1 for Kladno)

Mass size distributions of NMAHs are shown in Fig. 1 and S6. PM_1_ accounts for 80–90% of NCs, 70–80% of NSAs (as well as the NMAHs in total) and 40–60% of NPs. For all NMAH substance classes, the significance of the smallest size, PM_0.49_, was higher in summer than that in winter (in Ostrava). In contrast, the significance of a super-μm mode (3–7 μm) of NP and NSA MSDs decreased in summer, completely in the case of NSAs. A high fraction of NPs, 30–50%, was associated with the coarse fraction (PM_10_–PM_3_) in winter (Fig. 1). These results are in agreement with previous reports from other urban sites in central and southern Europe (Kitanovski et al. 2020) and China (Li et al. 2016). The aerosol number size distributions (characterised in Fig. S5) indicated close combustion sources and are consistent with the possible influence of wood burning. The MSDs peaking in the sub-micrometre size range highlight the significance of NMAHs’ inhalation exposure of the deep lung (Kitanovski et al. 2020), similar to other aromatic combustion byproducts like the parent PAHs (Ringuet et al. 2012) and polychlorinated dibenzodioxins and -furans (Zhang et al. 2016).

### Bioaccessibility

**Fig. 2:**
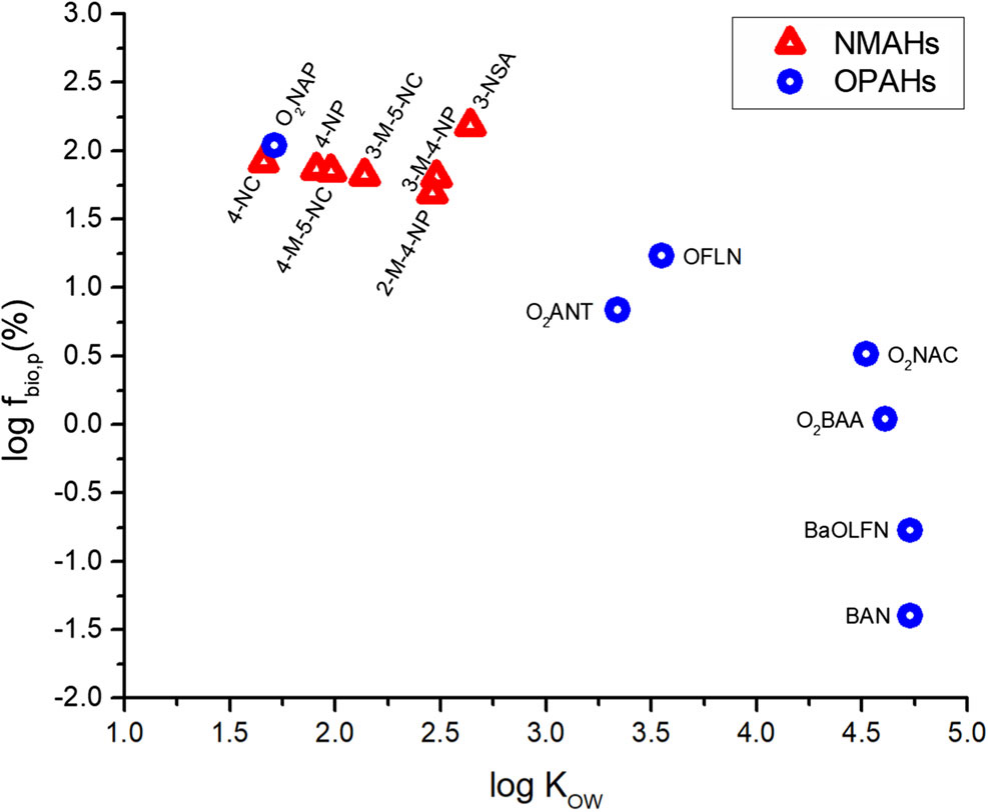
Fractions (%) of bioaccessible particulate mass of individual NMAHs and 7 oxygenated polycyclic aromatic hydrocarbons (OPAHs; Lammel et al. 2020b) varying with *K*_OW_, as addressed by simulated epithelial lung fluid ALF in PM_3_ samples collected in Ostrava winter. 3-NSA, 3-nitrosalicylic acid; 3-M-4-NP, 3-methyl-4-nitrophenol; 2-M-4-NP, 2-methyl-4-nitrophenol; 3-M-5-NC, 3-methyl-5-nitrocatechol; 4-M-5-NC, 4-methyl-5-nitrocatechol; 4-NP, 4-nitrophenol; 4-NC, 4-nitrocatechol; OFLN, 9-fluorenone; O_2_ANT, 9,10-anthraquinone; O_2_NAP, 1,4-naphthoquinone; O_2_NAC, 5,12-naphthacenequinone; O_2_BAA, benzanthracene-7,12-dione; BAN, benzanthrone; BaOFLN, benz(a)fluorenone

The lowermost 4 impactor stage filters of the campaigns at Kladno (1 winter-time sample set) and Ostrava (3 winter- and 3 summer-time sample sets), encompassing PM_3_, were leached in ALF. Only one sample set encompassing PM_3_ per location and season (3 sample sets in total) was leached in Gamble’s solution (GS; Table 2; Table S5; Fig. 2).

**Table 2:** Fractions (%) of the PM_3_ (PM_1_) size fractions being leached in simulated lung fluids, *f*_bio_p_ = *c*_p LLF_/*c*_p MeOH_, i.e. (a) artificial lysosomal fluid (ALF, pH 4.5) and (b) Gamble’s solution (GS, pH 7.4). One impactor sample per campaign

	Kladno winter	Ostrava winter	Ostrava summer
(a) ALF			
Σ_12_NMAH	55 (54)	75 (75)	94 (81)
Σ_2_NSA	95 (90)	123 (124)	140 (115)
Σ_4_NC	58 (54)	78 (75)	92 (79)
Σ_4_NP	45 (42)	66 (67)	83 (68)
(b) GS			
Σ_12_NMAH	66 (68)	56 (55)	64 (52)
Σ_2_NSA	119 (119)	101 (103)	114 (112)
Σ_4_NC	68 (66)	50 (49)	59 (55)
Σ_4_NP	72 (75)	77 (78)	64 (63)

Using ALF, more than half of NMAH mass was found bioaccessible in winter, and almost complete, 94%, in summer (Table 2a). This could be related to a higher content of hydrophobic substances in PM in winter. In central Europe, fossil fuel combustion byproducts, in particular PAHs, are much higher concentrated in winter, also in urban air, and also in Ostrava (Lammel et al. 2010; CHMI 2013; Vossler et al. 2015). The difference of *f*_bio_p_ found when using ALF across the campaigns (Table 2) was not pronounced as compared with when using GS to leach samples (insignificant differences for *p* < 0.05, *t* test). Often lower *f*_bio_p_ was found for all NMAH species when using the neutral GS than when using the acidic ALF (Table 2 and Table S5a; note that due to less samples leached by GS than ALF, directly comparable *f*_bio_p_ data are given in Table 2, but not in Table S5), but also the opposite was found (Kladno sample, Table 2, Table S5b). NSAs were almost completely bioaccessible, i.e. *f*_bio_p_≈100% in both LLFs. Bioaccessible fractions > 100% most likely reflect leaching procedure artefacts. They are more pronounced for NSAs and NCs when leached in ALF (Table S5). Therefore, we investigated the stability of NMAHs during the leaching procedure by spiking the LLFs with NMAH standard mix and carrying out the usual 24-h leaching. The results from stability study (Table S7) showed > 100% recoveries for NSAs in both LLFs, but usually < 100% recoveries for NCs. Neither NCs nor methylnitrophenols (MNPs) or dinitrophenols (DNPs) were found more stable in ALF than in GS (not significant, *p* < 0.05, *t* test). With a *pK*a (acidity constant) of 6.78 at 35 °C (Gelb et al. 1989), the majority of 4-NC (but also MNC) molecules will be deprotonated in GS (pH 7.4) at 37 °C. In deprotonated form, NCs are more susceptible to oxidation (e.g. by the dissolved oxygen in LLFs) and formation of nitrated 1,2-benzoquinones, which could not be measured by the analytical method employed here. In ALF at pH 4.5, NCs are in neutral form and more stable, hence their higher recoveries from ALF. This could also explain their higher *f*_bio_p_ in ALF (significant at the *p* < 0.05 level, *t* test; Table S5). For MNPs and DNPs, however, their lower stability in ALF is unexplained, having in mind their *pK*a values around 7.3 and 4.0, respectively (Schwarzenbach et al. 1988), as well as their high recoveries during the SPE clean-up after the leaching process (Table S3). Only for summer samples from Ostrava, the NMAHs’ bioaccessible fractions in ALF are much higher than 100% (range, 99–187%; Table S5b), suggesting the possible aqueous-phase formation of NMAHs from their precursors in the PM during the leaching process (positive artefact) under mild acidic conditions (pH 4.5; Kroflič et al. 2018). This hypothesis is supported by the high levels of PM_10_ (PM_2.5_), NO_x_ and Fe (Fig. S3) measured during the summer sampling campaign (Table 1a) which could facilitate the oxidation and nitration of NMAH precursors. Interestingly, for the same sample sets, very low bioaccessibility in GS was observed for NCs (range, 9–77%; Table S5b) that cannot be solely explained by the NC stability results (50–99%; Table S7). Due to high Fe content in samples, NCs could partly exist as monocomplexes of Fe^3+^ and enhance the production of reactive species by Fenton or Fenton-like systems (Salgado et al. 2017). During these processes, NCs can be oxidised or degraded by the formed reactive species, thus diminishing their leached concentrations (negative artefact), as well as their measured bioaccessible fractions.

For both LLFs, *f*_bio_p_ was found independent of particle size, i.e. do not differ significantly between sub-micrometre particles and the PM_3_ size fraction (*p* < 0.05, *t* test; Table 2). This is also reflected as similar (statistically not different, *p* < 0.05) values for PM_1_/PM_3_ found for PM methanol extracts as for LLFs (Table S6).

The range of physicochemical properties of NMAHs is not large, 2 and 1 order of magnitude for water solubility, *s*, and *K*_OW_, respectively (listed in Table S2). The respective data for Ostrava winter (Table S5) are shown together with data for 7 oxygenated polycyclic aromatic hydrocarbons (OPAHs; Lammel et al. 2020b), hence, s and K_OW_ across the two substance classes ranging 5 and 4 orders of magnitude, respectively (Fig. 2, Fig. S7). The bioaccessible fractions of NMAHs, *f*_bio p_, were similar in winter and summer (Fig. S7), reflecting that ambient aerosol chemical composition in source areas (anthropogenic sources) is subject to little seasonal variation (Putaud et al. 2010). It decreased with the compound’s increasing *K*_OW_ (Fig. 2) and decreasing water solubility (Fig. S7). Bioaccessibility may be negligible for lipophilic substances (i.e. log *K*_OW_ > 4.5). A lack of a clear trend in Fig. S7 reflects the aqueous electrolyte nature of the LLFs.

The MSDs of the bioaccessible fractions were only slightly shifted against the MSDs of the PM methanol extracts. For example, for GS, the bioaccessible sub-micrometre mass fraction in PM_3_, i.e. PM_1_/PM_3_, deviated typically only within 2% from the total sub-micrometre mass fraction in PM_3_ (Table S6b), while for ALF these shifts were up to ≈ 10% (Table S6a), in the sense that the sub-micrometre fraction was less bioaccessible than the coarse size fraction. This is possibly related to a higher hydrophobicity of PM_1_ particles as compared with coarse PM. Hydrophobicity may limit the leachability of particles. Hydrophobicity was not determined, but more than 60% of EC and OC, which often represent hydrophobic constituents, were associated with the PM_1_ mass fraction, more than in coarse PM (cumulative MSDs, Fig. S5).

## Conclusions and suggestions for research

Inhalation bioaccessibility of the nitrated monoaromatic pollutants in PM as operationally defined by leaching filter samples in simulated lung fluids was found very high for both an aqueous acidic (pH 4.5, ALF) and a neutral electrolyte (pH 7.4, Gamble’s solution). This emphasises the human inhalation exposure to polar constituents of particulate organic matter. Bioaccessibility of a given PM constituent will depend on not only the substance properties but also the aerosol matrix (e.g. its hydrophobicity). Here, a limited number of samples have been analysed. Among aerosol types, only urban aerosols, strongly influenced by fossil fuel burning sources (metallurgical industries and coal production and burning, road traffic; Lammel et al. 2020b) were covered. More such data should be gained from other aerosol types and extended to other organic pollutants, abundant in aerosols, such as polycyclic aromatic compounds. The determination of bioaccessibility based on leaching with simulated lung fluids may even be an underestimate, as ultrafine particles may penetrate through the membrane and thus deliver pollutants without dissolution in the lung fluid. On the other hand, the presence of false-positive (*f*_bio p_ >> 100%) and false-negative artefacts (*f*_bio p_ < 50%) during the in vitro tests of bioaccessibility should be avoided by (a) optimization of the duration of the tests (allowing less time for unwanted reactions to occur), (b) using degassed LLFs and performing the tests in inert atmosphere for analytes that could be easily oxidised (which is opposite to the real conditions in the lung) and (c) by using more realistic LLF models that contain lipids, proteins and antioxidants (e.g. Boisa et al. 2014). The presence of organic constituents and antioxidants in LLFs would serve as “buffer” for PM and potentially in situ formed ROS during the leaching procedure. Only the bioaccessible fraction of pollutants can become biologically effective, such as ROS active. While the reduction potential as an indicator for redox reactivity is available for a number of NMAHs such as nitrobenzenes (Uchimiya et al. 2010), determination of the oxidative potential (OP) of organic pollutants has so far been limited to quinones (Charrier and Anastasio 2012; Yu et al. 2018; Lammel et al. 2020b) and N-heterocycles (Dou et al. 2015). Finally, the inhalation exposure to the targeted NMAHs is in fact higher, because part of the NMAH mass will be distributed to the gas-phase of ambient aerosols, not considered in this study.

## Electronic supplementary material

ESM 1 (PDF 860 kb)
