# Supplementary material for: Nitrated monoaromatic hydrocarbons (nitrophenols, nitrocatechols, nitrosalicylic acids) in ambient air: levels, mass size distributions and inhalation bioaccessibility
Source: Environ Sci Pollut Res Int. 2020 Jun 11;28(42):59131–40. doi: 10.1007/s11356-020-09540-3 (PMC8541976; doi:10.1007/s11356-020-09540-3)
Supplement: Supplementary file 1 — (PDF 860 kb) [file 11356_2020_9540_MOESM1_ESM.pdf]

Supplementary Material

Nitrated monoaromatic hydrocarbons (nitrophenols, nitrocatechols, nitrosalicylic acids) in ambient air - levels, phase partitioning, mass size distributions and inhalation bioaccessibility

Zoran Kitanovski[1], Jan Hovorka[2], Jan Kuta[3], Cecilia Leoni[2], Roman Prokeš[3], Ondřej Sáňka[3], Pourya Shahpoury[1,4], Gerhard Lammel[1,3]

[1] Max Planck Institute for Chemistry, Multiphase Chemistry Department, Mainz, Germany
[2] Charles University, Faculty of Science, Institute for Environmental Studies, Prague, Czech Republic
[3] Masaryk University, Research Centre for Toxic Compounds in the Environment, Brno, Czech Republic
[4] Environment and Climate Change Canada, Air Quality Processes Research Section, Toronto, Canada

S1 Methods

S1.1 Sites

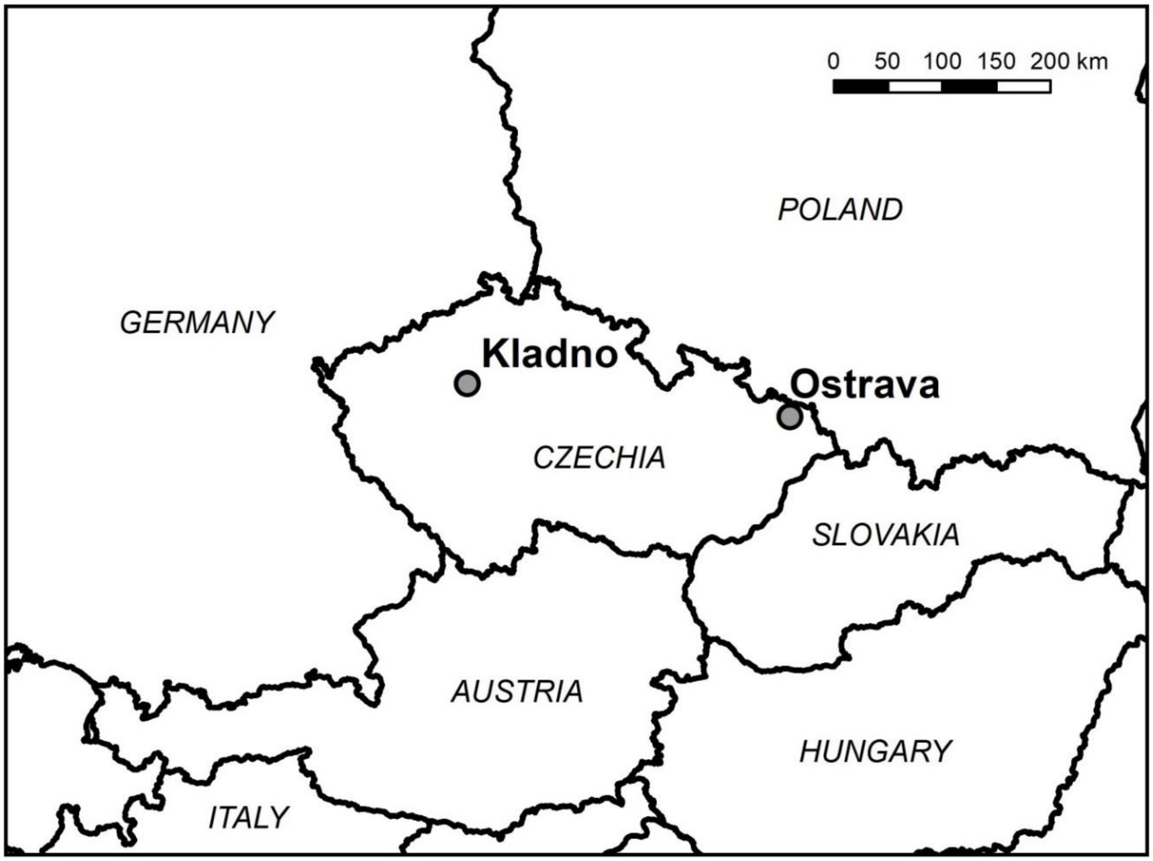

Fig. S1 Location of sites

S1.2 Chemicals

Table S1. Composition of simulated lung fluids (mg L^{-1}) used i.e., artificial lysosomal fluid (ALF; Colombo et al., 2008) and Gamble's solution (GS; Marques et al., 2011).

	ALF	GS
Sodium chloride	3210	6019
Calcium chloride dihydrate	128	368
Magnesium chloride	50	95
Disodium hydrogen phosphate	71	126
Potassium chloride	-	298
Sodium sulfate	39	63
Sodium hydroxide	6000	-
Sodium acetate	-	574
Sodium hydrogen carbonate	-	2604
Sodium tartrate dihydrate	90	-
Sodium pyruvate	86	-
Sodium lactate	85	-
Citric acid	20800	-
Sodium citrate dihydrate	77	97
Glycine	59	-
pH	4.5	7.4
Ionic strength	0.14 M	0.12 M

Table S2. Targeted substance, physico-chemical properties vapour pressure (p), water solubility (s) and octanol-water partitioning coefficient (K_{ow}) at 298 K (some s at 293 K). n.d. = no data.

Analyte	Abbreviation	Supplier	p (µPa)[e,m]	s (mg L^{-1})	log K_{ow}
3-Nitrosalicylic acid	3-NSA	Sigma-Aldrich	1.5	1300 [y]	2.64 [c]
5-Nitrosalicylic acid	5-NSA	Sigma-Aldrich	0.17	2000 [y]	n.d.
4-Nitrocatechol	4-NC	Sigma-Aldrich	5.96	10500 [e]	1.66 [c]
4-Nitroguaiacol (2-Methoxy-4-nitrophenol)	4-NG	Sigma-Aldrich	248	2055 [e]	1.73 [c]
4-Methyl-5-nitrocatechol	4-M-5-NC	Santa Cruz Biotechnologies	9.75	4853 [e]	1.98 [e]
4-Nitrophenol	4-NP	Sigma-Aldrich (>96%)	385	11600 [s]	1.91 [c]
2,4-Dinitrophenol	2,4-DNP	Sigma-Aldrich	n.d.	2790 [s]	n.d.
3-Methyl-4-nitrophenol	3-M-4-NP	Sigma-Aldrich	9.75	1190 [s]	2.48 [c]
3-Methyl-5-nitrocatechol	3-M-5-NC	Chemhere Co. Ltd (>95%)	n.d.	n.d.	2.14 [a]
2-Methyl-4-nitrophenol	2-M-4-NP	Sigma-Aldrich	2.25	2235 [e]	2.46 [e]
2-Methyl-4,6-dinitrophenol (4,6-Dinitro-ortho-cresol)	DNOC	Sigma-Aldrich	629	6940 [b]	2.46 [e]

[a] estimate, ACDLabs, 2015
[b] BCPC, 2012
[c] experimental value, ChemSpider, 2019
[e] estimate EPIsuite, USEPA, 2012
[m] modified Grain method
[s] Schwarzenbach et al., 1988
[y] Myrdal et al., 1992

Table S3. Solid-phase extraction (SPE) recoveries of targeted analytes, extracted from simulated lung fluids (artificial lysosomal fluid and Gamble's solution). SPE recovery study was done according to the post-extraction spike method described by Matuszewski et al. (2003).

Analyte	SPE recovery (%)[a]	
	ALF	Gamble's solution
3-NSA	96.6±10.9[b]	95.2±11.6[b]
5-NSA	95.0±6.6	94.9±10.7
4-NP	100.5±0.8	99.8±0.2
4-NG	101.2±2.7	101.4±7.7
3-M-4-NP	100.5±1.8	99.3±1.9
2-M-4-NP	100.6±2.7	99.6±1.1
DNOC	105.1±8.2	104.1±60.3
2,4-DNP	103.1±5.5	100.5±25.7
4-NC	97.8±11.0	98.4±11.3
4-M-5-NC	96.6±7.0	96.0±9.5
3-M-5-NC	95.5±7.8	96.4±10.5

[a] n=3

[b] mean recovery ± standard deviation

S1.3 Chemical analysis

Table S4. Field blank based LOQs of NMAHs in (a) methanol extracts and (b) LLF leachates per impactor stage for various campaigns (pg m^{-3}).

a)

Analyte	Kladno / Ostrava winter 2016	Ostrava summer 2016
3-NSA	6.2[a]	6.5[a]
5-NSA	6.2[a]	6.5[a]
4-NP	6.2[a]	6.5[a]
4-NG	61[a]	26[a]
3-M-4-NP	6.2[a]	6.5[a]
2-M-4-NP	6.2[a]	6.5[a]
DNOC	6.0[a]	6.5[a]
2,4-DNP	6.0[a]	6.5[a]
4-NC	6.2[a]	6.5[a]
4-M-5-NC	6.2[a]	6.5[a]
3-M-5-NC	6.2[a]	6.5[a]
3-M-4-NC	6.2[a]	6.5[a]

b)

Analyte	Kladno / Ostrava winter 2016	Ostrava summer 2016
	ALF / GS	ALF / GS
3-NSA	6.2[a] / 10[b]	6.7 / 3.4[a]
5-NSA	6.2[a] /10[b]	6.7 / 3.4[a]
4-NP	6.2[a] / 10[b]	6.7 / 3.4[a]
4-NG	12.4[a] / 20[b]	13.4 / 7.0[a]
3-M-4-NP	6.2[a] / 10[b]	6.7 / 3.4[a]
2-M-4-NP	6.2[a] / 10[b]	6.7 / 3.4[a]
DNOC	6.1[a] / 10[b]	6.7 / 3.4[a]
2,4-DNP	6.2[a] / 10[b]	6.7 / 3.4[a]
4-NC	6.2[a] / 10[b]	6.7 / 3.4[a]
4-M-5-NC	6.2[a] / 10[b]	6.7 / 3.4[a]
3-M-5-NC	6.2[a] / 10[b]	6.7 / 3.4[a]
3-M-4-NC	6.2[a] / 10[b]	6.7 / 3.4[a]

[a]4 times higher for the backup filter i.e., $PM_{0.49}$
[b]2 times higher for the backup filter i.e., $PM_{0.49}$

S.1.4 Other supporting parameters

Meteorological (temperature, humidity, wind) and trace gases i.e., CO, O_3, SO_2, NO and NO_2, were routinely monitored at the Ostrava station and provided by the Czech Hydrometeorological Institute, while at Kladno, these measurements were provided by the Air Quality Laboratory station of the Charles University.

QFFs were weighed using a laboratory balance following accommodation to a constant humidity and temperature. PM total element and carbon fraction concentrations were determined from punches of baked quartz filters. Carbon mass fractions were thermally desorbed from the filter medium under a He atmosphere followed by an oxidizing atmosphere using an OC-EC Aerosol Analyzer, Sunset Laboratory, Tigard, USA. A flame ionization detector (FID) was used to monitor the CO_2 concentration. For heating ramps, the EUSAAR-2 protocol (Cavalli and Putaud, 2008) was followed.

The content of selected metals (Ti, V, Cr, Mn, Fe, Co, Ni, Cu, Zn, Mo, Cd and Pb) was determined after microwave digestion (MWS 3+ Berghof system, Germany) of samples with concentrated nitric acid (4 mL, Merck p.a.) and hydrogen peroxide (2 mL, Merck p.a.) followed by inductively coupled plasma mass spectrometry (Agilent 7700x ICP-MS, Japan). External calibration with internal standard correction (^{45}Sc, ^{72}Ge, ^{103}Rh and ^{209}Bi) was applied for quantification of elements. The analytical method was validated using samples spiked with known amount of analytes and recoveries were found within the range 90–110 %.

Particle mass size distributions (MSDs) were derived assuming sphericity and a density of 2 g cm^{-3} from number size distributions. Particle counting at Kladno was done using a scanning mobility particle sizer (80 channels in the range 0.016-0.74 µm) in combination with an aerodynamic particle sizer (52 channels in the range 0.5-20 µm). The whole size range spectra were merged into spectra of particle number or mass versus aerodynamic particle diameter. In Ostrava, size spectra were determined using a Palas Fidas 200S OPC with 250 channels in the range 0.15-18 µm.

S2. Results

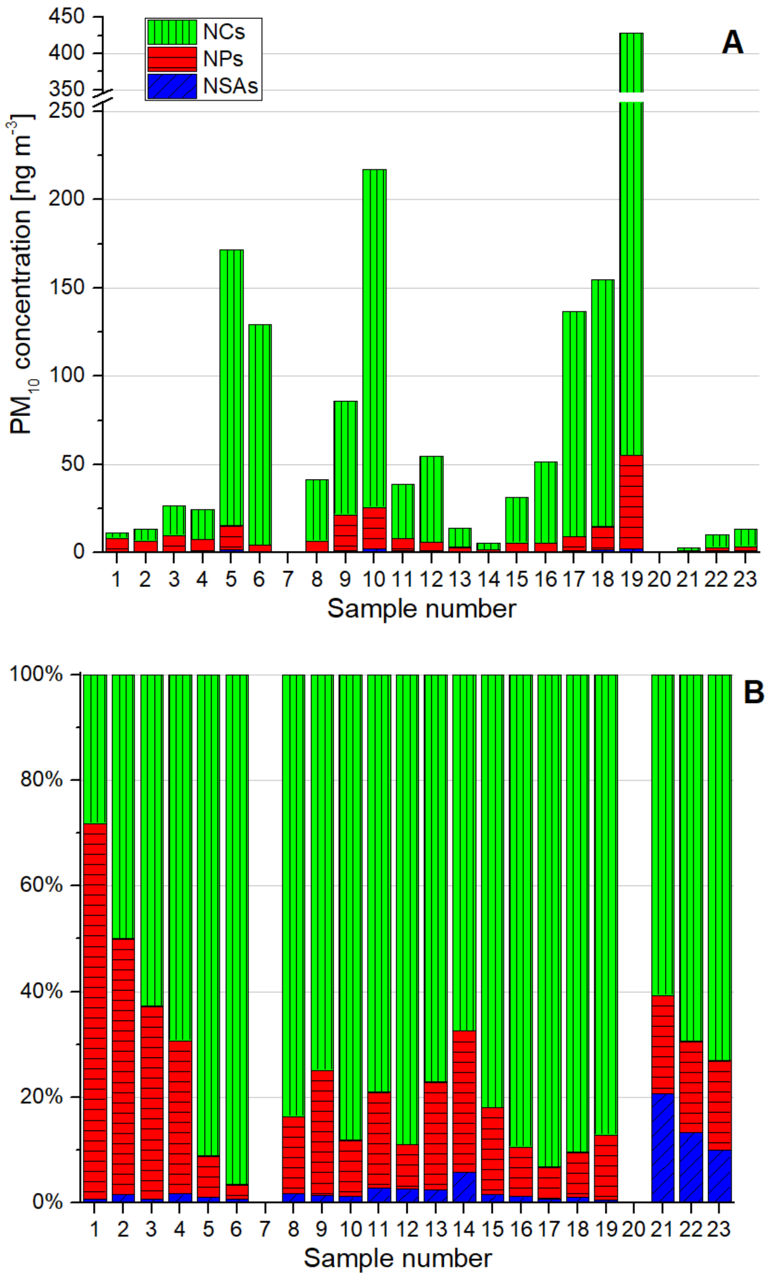

Fig. S2 Temporal variation of NMAH PM_{10} concentrations (ng m^{-3}) during the campaigns Kladno winter (subsequent 12-h and 24-h samples no. 1-6), Ostrava winter (24-h samples no. 8-19), and Ostrava summer (96-h samples no. 21-23), (A) absolute (ng m^{-3}) and (B) relative (%). NCs = nitrocatechols (sum of 4-NC, 4-M-5-NC, 3-M-5-NC and 3-M-4-NC), NPs = nitrophenols (sum of 4-NP, 2-M-4-NP, 3-M-4-NP, 4-NG, DNOC and 2,4-DNP), NSAs = nitrosalicylic acids (sum of 3-NSA and 5-NSA)

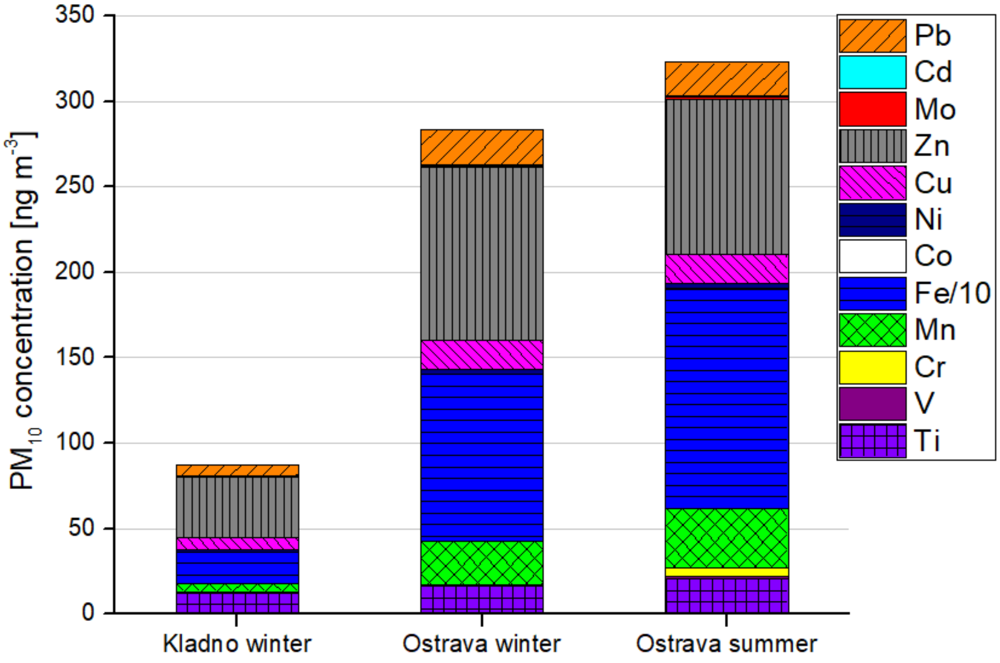

Fig. S3 Transition metals and lead average PM_{10} concentrations and patterns (ng m^{-3}, except Fe: divided by10 (in ng m^{-3}))

a)

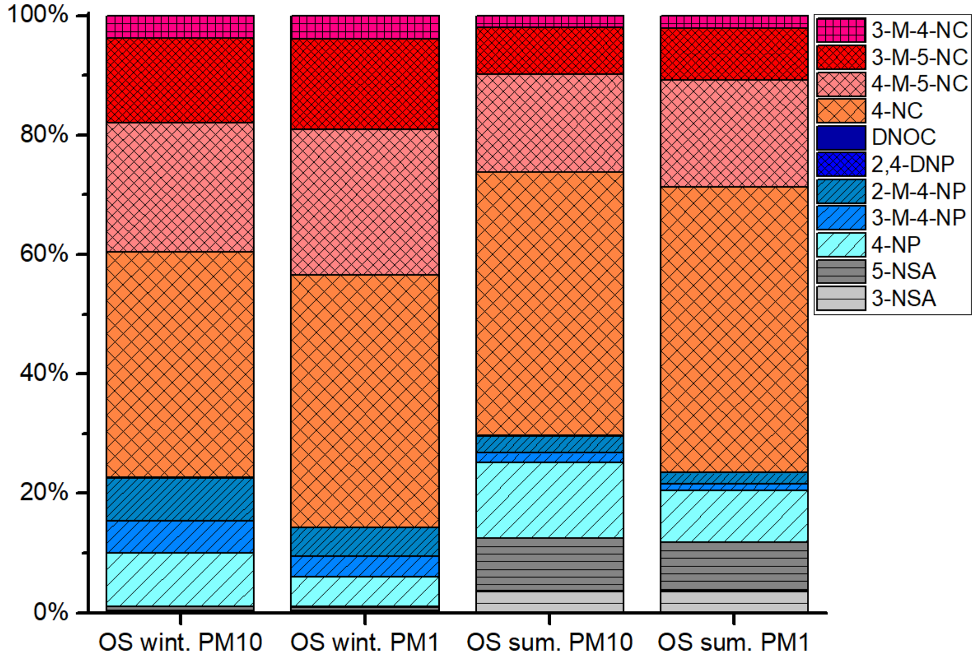

b)

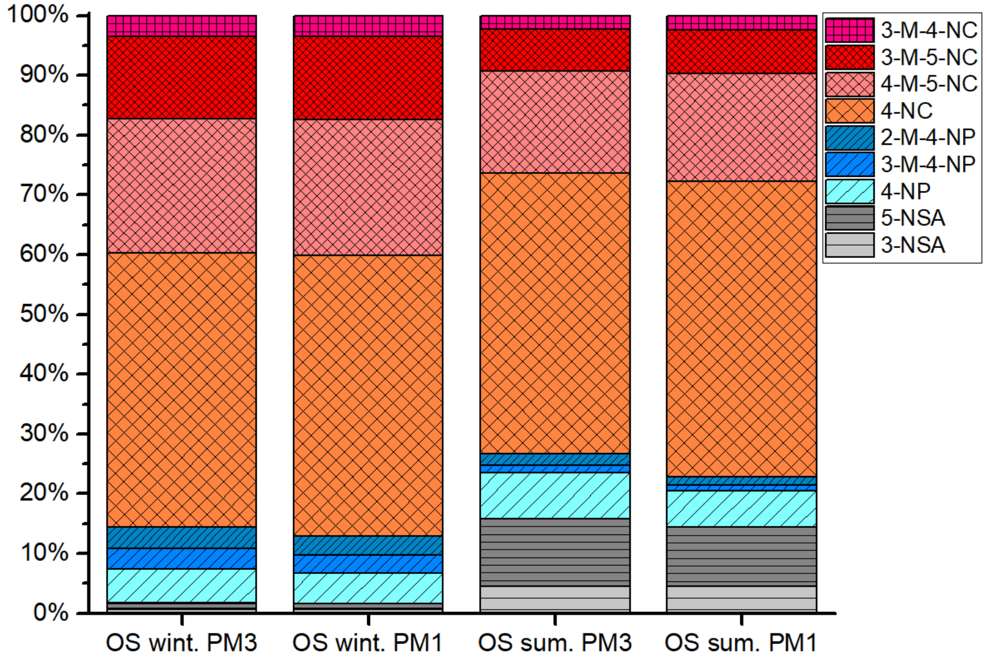

Fig. S4 Substance patterns of NMAHs in (a) PM_{10} and PM_1 (methanol extract) and (b) bioaccessible PM_3 and PM_1 fraction (ALF leachate), across seasons. OS = Ostrava, wint. = winter, sum. = summer

<table>
<tr><td></td><td></td><td>PM</td><td>C class mass</td></tr>
<tr><td>Kladno
winter</td><td>N = 2.2×10^{4} cm^{-3}
S = 1.9×10^{-5} cm^{-1}
V = 5.1×10^{-10}</td><td>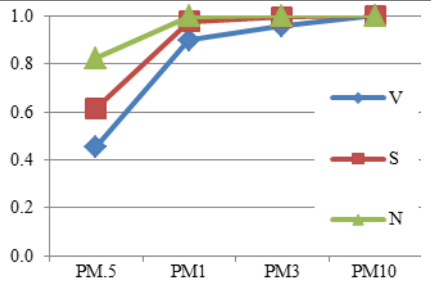
</td><td>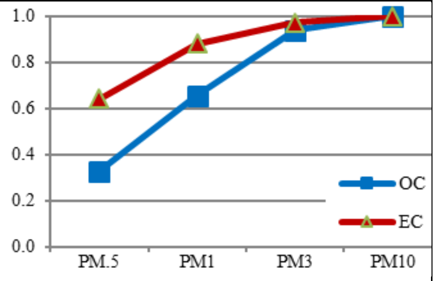
</td></tr>
<tr><td>Ostrava
winter</td><td>N = 6.5×10^{4} cm^{-3}
S = 6.2×10^{-4} cm^{-1}
V = 8.4×10^{-9}</td><td>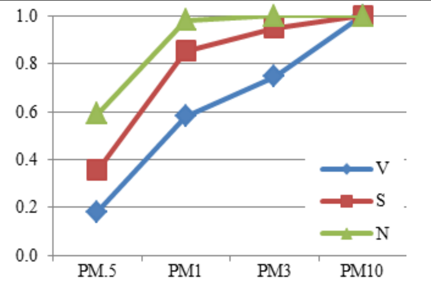
</td><td>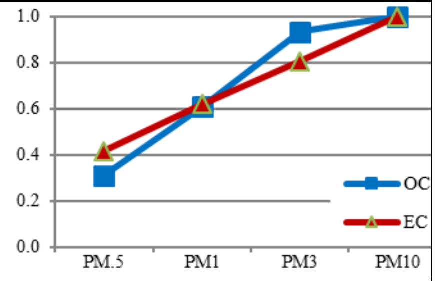
</td></tr>
<tr><td>Ostrava
summer</td><td>N = 2.8×10^{4} cm^{-3}
S = 2.5×10^{-4} cm^{-1}
V = 4.1×10^{-9}</td><td>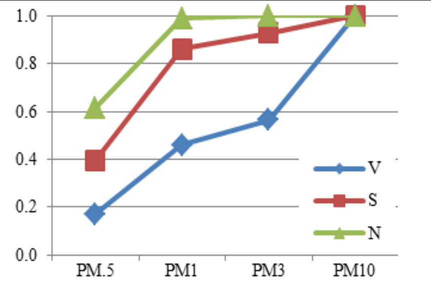
</td><td>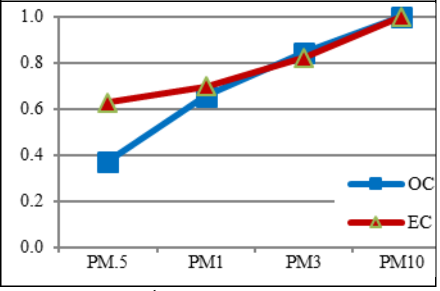
</td></tr>
</table>

Fig. S5 Time-weighted mean total aerosol number N (cm^{-3}), surface S (cm^{-1}) and volume V (cm^{3} cm^{-3}) concentrations and corresponding cumulative mass size distributions of PM and OC and EC. Kladno: D = 0.3-10 µm; Ostrava: D = 0.3-35 µm.

	NMAH	TM
Kladno winter	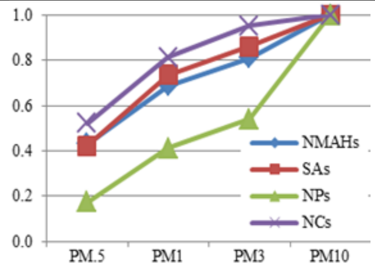	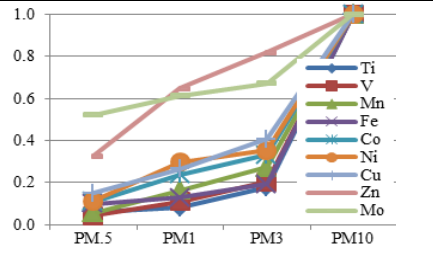
Ostrava winter	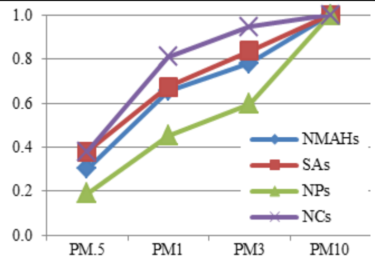	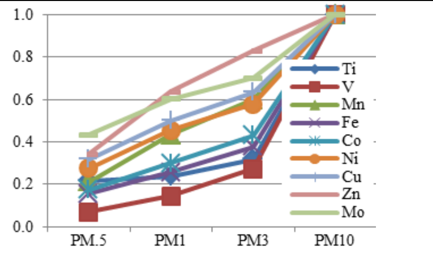
Ostrava summer	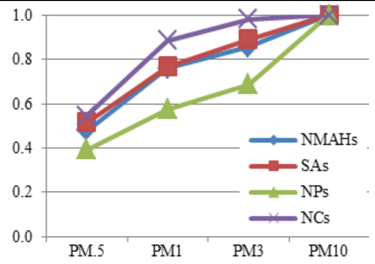	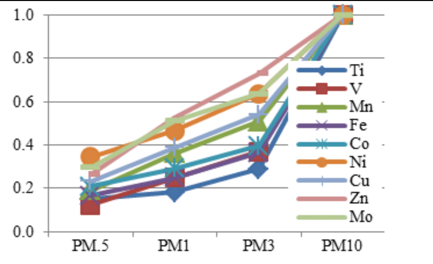

Fig. S6 Time-weighted mean cumulative mass size distributions (MSDs) of NMAHs' and transition metals' (TM) mass fractions of PM size fractions at various sites and seasons

Table S5. Bioaccessible fractions $f_{bio} = c_{p\ LLF} / c_{p\ MeOH}$ (%) in PM_3 mobilized by simulated lung fluids, (a) mean ± standard deviation, and (b) means across campaigns. ALF = artificial lysosomal fluid (pH 4.5); GS = Gamble's solution (pH 7.4); n.d. = no data.

a)

Analyte	ALF (n = 7)	GS (n = 3)
3-NSA	145 ± 27	97 ± 12
5-NSA	123 ± 57	109 ± 11
4-M-5-NC	102 ± 50	49 ± 12
3-M-5-NC	94 ± 62	35 ± 9
3-M-4-NC	86 ± 44	9 ± 5
4-NC	104 ± 52	70 ± 8
4-NP	73 ± 23	64 ± 5
4-NG	n.d.[a]	n.d.[a]
DNOC	n.d.[a]	n.d.[a]
2,4-DNP	n.d.[a]	n.d.[a]
3-M-4-NP	81 ± 39	82 ± 3
2-M-4-NP	70 ± 40	77 ± 4

b)

Analyte	ALF			GS		
	Kladno winter (n = 1)	Ostrava winter (n = 3)	Ostrava summer (n = 3)	Kladno winter (n = 1)	Ostrava winter (n = 1)	Ostrava summer (n = 1)
3-NSA	114	156	164	106	83 [b]	101 [b]
5-NSA	78	105	187	107	98 [b]	120 [b]
4-M-5-NC	68	77	160	63	43 [b]	41 [b]
3-M-5-NC	53	64	166	45	30 [b]	30 [b]
3-M-4-NC	59	62	136	14	5 [b]	9 [b]
4-NC	65	84	163	72	61 [b]	77 [b]
4-NP	54	67	99	68	66 [b]	59 [b]
4-NG	n.d.[a]	n.d.[a]	n.d.[a]	n.d.[a]	n.d.[a]	n.d.[a]
DNOC	n.d.[a]	n.d.[a]	n.d.[a]	n.d.[a]	n.d.[a]	n.d.[a]
2,4-DNP	n.d.[a]	n.d.[a]	n.d.[a]	n.d.[a]	n.d.[a]	n.d.[a]
3-M-4-NP	55	63	127	79	83 [b]	85 [b]
2-M-4-NP	43	52	116	74	81 [b]	74 [b]

[a] because of most $c_{p\ MeOH}$ <LOQ

[b] 1 out of 3 impactor samples only

Table S6. Sub-micrometer mass fraction in PM_3 i.e., PM_1/PM_3 (%) in methanol extract and bioaccessible mass fraction (separated by '/') based on (a) ALF and (b) GS leachates. ALF = artificial lysosomal fluid (pH 4.5), GS = Gamble's solution (pH 7.4), n = number of impactor samples.

a)

	Methanol extract / ALF		
	Kladno winter n = 1	Ostrava winter n = 3	Ostrava summer n = 3
Σ_2NSA	83 / 78	78 / 81	81 / 72
Σ_4NC	84 / 82	83 / 84	89 / 83
Σ_5NP	72 / 68	71 / 73	74 / 58
Σ_{11}NMAH	74 / 81	72 / 82	80 / 79

b)

	Methanol extract / GS		
	Kladno winter n = 1	Ostrava winter n = 1	Ostrava summer n = 1
Σ_2NSA	83 / 83	76 / 77	82 / 81
Σ_4NC	84 / 86	85 / 84	90 / 89
Σ_5NP	72 / 74	72 / 73	74 / 73
Σ_{11}NMAH	74 / 85	75 / 81	82 / 86

Table S7. NMAH stability in artificial lysosomal fluid (ALF) and Gamble's solution during the leaching process (24 h shaking at 60 rpm, at 37°C, in the dark).

Analyte	NMAH recovered (%)[a]	
	ALF	Gamble's solution
3-NSA	135.2±3.5[b]	139.6±6.2[b]
5-NSA	134.3±0.6	128.6±2.6
4-NP	110.6±1.6	113.7±0.5
4-NG	83.7±0.6	86.1±1.2
3-M-4-NP	78.2±0.4	90.4±0.2
2-M-4-NP	77.2±0.5	94.3±0.6
DNOC	45.6±4.1	67.5±2.0
2,4-DNP	76.2±3.6	89.2±2.8
4-NC	119.7±2.9	99.0±1.3
4-M-5-NC	90.9±2.3	66.9±5.0
3-M-5-NC	74.0±2.4	49.5±6.2

[a] n=3

[b] mean recovery ± standard deviation

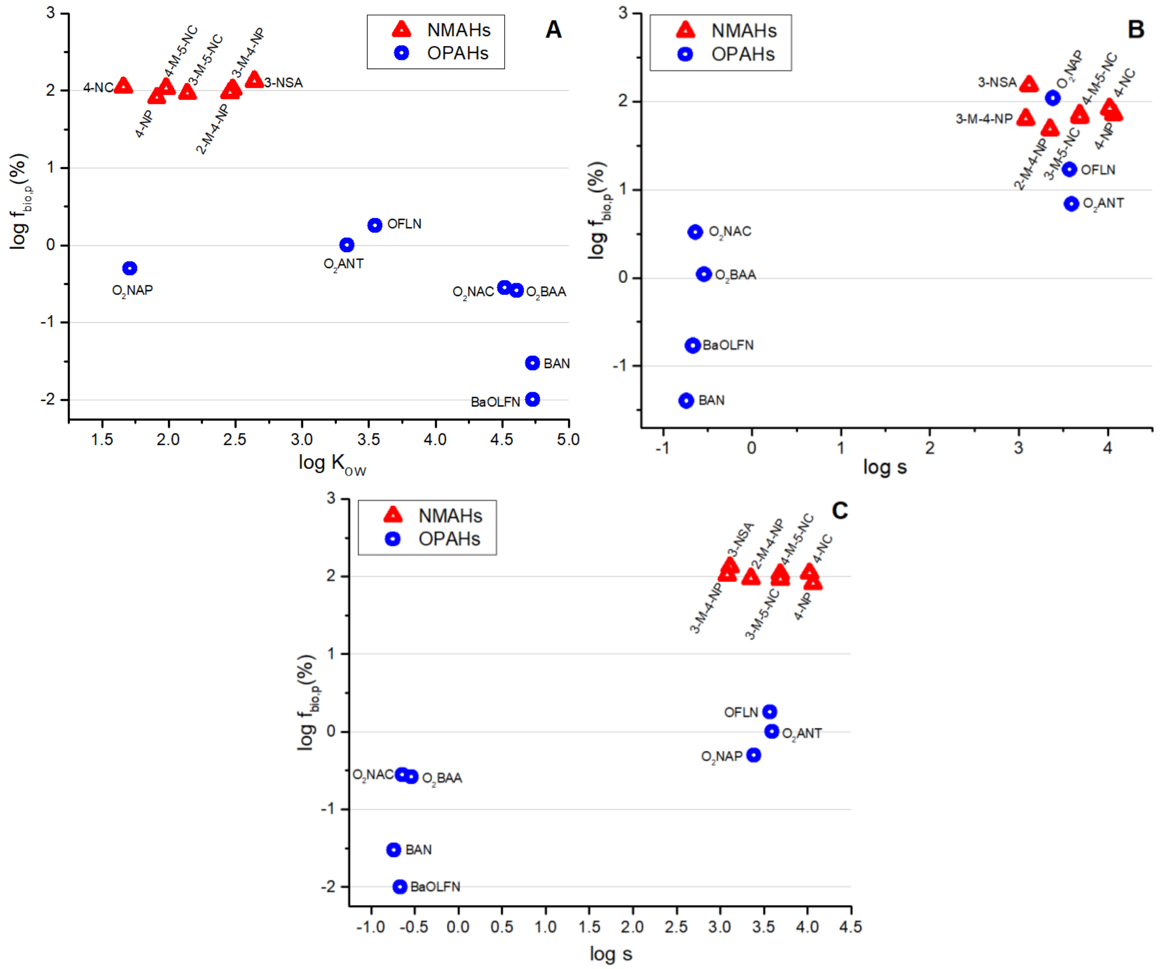

Fig. S7. Fractions (%) of bioaccessible particulate mass of individual NMAHs and 7 oxygenated polycyclic aromatic hydrocarbons (OPAHs; Lammel et al., 2020) varying with (a) K_{OW} in summer, (b) water solubility, s, in winter and (c) s in summer, as addressed by simulated lung fluid ALF in PM_3 samples collected in Ostrava. 3-NSA=3-nitrosalicylic acid; 3-M-4-NP=3-methyl-4-nitrophenol; 2-M-4-NP=2-methyl-4-nitrophenol; 3-M-5-NC=3-methyl-5-nitrocatechol; 4-M-5-NC=4-methyl-5-nitrocatechol; 4-NP=4-nitrophenol; 4-NC=4-nitrocatechol; OFLN=9-fluorenone; O_2ANT=9,10-anthraquinone; O_2NAP=1,4-naphthoquinone; O_2NAC=5,12-naphthacenequinone; O_2BAA=benzanthracene-7,12-dione; BAN=benzanthrone; BaOFLN=benz(a)fluorenone

Supplementary references

ACD/Labs. (2015) Absolv; Advanced Chemistry Development Inc., Toronto, Canada.

BCPC, 2012. The pesticide manual, British Crop Production Council, MacBean C (ed.), 16th ed., BCPC Publications, Alton, UK, 1439 pp.

Cavalli F, Putaud JP (2008) Toward a standardized thermal-optical protocol for measuring atmospheric organic and elemental carbon: The EUSAAR protocol. Report #EUR 23441 EN, Office for Official Publications of the European Communities, Luxembourg, 29 pp.

ChemSpider chemical structure database, 2019. Royal Society of Chemistry, URL: http://www.chemspider.com/ (last time accessed 20 November 2019)

Colombo C, Monhemius AJ, Plant JA (2008) Platinum, palladium and rhodium release from vehicle exhaust catalysts and road dust exposed to simulated lung fluids. Ecotox Environ Safety 71:722-730. https://doi.org/10.1016/j.ecoenv.2007.11.011

Lammel G, Kitanovski Z, Kukučka P, Novák J, Arangio A, Codling GP, Filippi A, Hovorka J, Kuta J, Leoni C, Přibylová P, Prokeš R, Sáňka O, Shahpoury P, Tong HJ, Wietzoreck M (2020) Levels, phase partitioning, mass size distributions and bioaccessibility of oxygenated and nitrated polycyclic aromatic hydrocarbons (OPAHs, NPAHs) in ambient air, Environ Sci Technol 54:2615-2625. https://dx.doi.org/10.1021/acs.est.9b06820

Marques MRC, Loebenberg R, Almukainzi M (2011) Simulated biological fluids with possible application in dissolution testing. Dissolution Technol. 15–28. https://doi.org/10.14227/DT180311P15

Matuszewski BK, Constanzer ML, Chavez-Eng CM (2003) Strategies for the assessment of matrix effect in quantitative bioanalytical methods based on HPLC-MS/MS. Anal Chem 75:3019-3030. https://doi.org/10.1021/ac020361s

Myrdal P, Ward GH, Dannenfelser RM, Mishra D, Yalkowsky SH (1992) AQUAFAC 1: Aqueous functional group activity coefficients; application to hydrocarbons. Chemosphere 24:1047-1061. https://doi.org/10.1016/0045-6535(92)90196-X

Schwarzenbach RP, Stierli R, Folsom BR, Zeyer J (1988) Compound properties relevant for assessing the environmental partitioning of nitrophenols. Environ Sci Technol 22:83-92, https://doi.org/10.1021/es00166a009

USEPA (2012) EPI-Suite. Estimation Programs Interface Suite for Microsoft®Windows, v 4.11; United States Environmental Protection Agency.
